# Human low-threshold mechanoafferent responses to pure changes in friction controlled using an ultrasonic haptic device

**DOI:** 10.1038/s41598-021-90533-8

**Published:** 2021-05-27

**Authors:** Mariama Dione, Roger Holmes Watkins, Eric Vezzoli, Betty Lemaire-Semail, Johan Wessberg

**Affiliations:** 1 Department of Physiology, Institute of Neuroscience and Physiology, Sahlgrenska Academy, University of Gothenburg, Gothenburg, Sweden; 2 Univ. Lille, Arts et Métiers Institute of Technology, Centrale Lille, Junia, ULR 2697‐L2EP, F-59000 Lille, France

**Keywords:** Somatic system, Peripheral nervous system, Sensory processing, Touch receptors, Neurophysiology

## Abstract

The forces that are developed when manipulating objects generate sensory cues that inform the central nervous system about the qualities of the object’s surface and the status of the hand/object interaction. Afferent responses to frictional transients or slips have been studied in the context of lifting/holding tasks. Here, we used microneurography and an innovative tactile stimulator, the Stimtac, to modulate both the friction level of a surface, without changing the surface or adding a lubricant, and, to generate the frictional transients in a pure and net fashion. In three protocols, we manipulated: the frictional transients, the friction levels, the rise times, the alternation of phases of decrease or increase in friction to emulate grating-like stimuli. Afferent responses were recorded in 2 FAIs, 1 FAII, 2 SAIs and 3 SAIIs from the median nerve of human participants. Independently of the unit type, we observed that: single spikes were generated time-locked to the frictional transients, and that reducing the friction level reduced the number of spikes during the stable phase of the stimulation. Our results suggest that those frictional cues are encoded in all the unit types and emphasize the possibility to use the Stimtac device to control mechanoreceptor firing with high temporal precision.

## Introduction

The forces that are developed when we manually interact with surfaces or objects present in our environment provide essential frictional cues that inform the central nervous system about the characteristics of the surface touched or, in the case of object manipulation, about the amount of force needed to secure the grip and avoid object slippage^1^.

When we explore surfaces with mediolateral movements of the finger^2–4^, or when we lift and hold small objects using the precision grip^5^, the forces change with the movement dynamics^6,7^, which reflects how the skin is deformed and stretched by the interaction^8,9^. Four main phases have been described: contact, loading, sliding/holding, unloading^5–7^. Under contact, the skin is just pressed on the surface (increase in pressure force). Under loading, the finger does not move, but the skin starts to stretch. Both lateral and pressure forces increase together. Then depending on the task, the finger either moves over the surface (sliding phase to explore the surface), or the fingers remain static to hold the object. In both cases, the forces are maintained at a stable high level. During sliding, the area of contact between the skin and the surface decreases while the skin is still under stretch^6,8,9^. Forces finally unload to release the object or end the movement exploration.

From the force interactions occurring during surface exploration or object manipulation, a number of frictional cues can be derived. During surface exploration, the friction coefficient, which is measured as the ratio between the lateral and normal forces during sliding, inform us about the surface’s characteristics such as its stickiness/slipperiness^10,11^, its roughness/smoothness^12^, or its pleasantness^13^. The friction coefficient and the intensity of the sensation are both reduced when the surface is lubricated with greasy and occlusive agents such as soap^12,14^ or with the skin moisture when the finger is very wet^15,16^. However, the coefficient of friction is increased under normal levels of skin moisture^15,17–19^. When the fingers slide over coarse surfaces such as raised dots, skin deformation that occurs during sliding induces small variations in the lateral force, which also informs us about the roughness of the surface^12,20^. When a very smooth surface such as glass is used, the movement is not completely smooth during sliding and has been characterized as a stick–slip motion, with series of microslips occurring^21^. Materials with very similar friction coefficients such as glass and plastic, may be readily distinguished by the mechanical events occurring before and during the transition between loading and slipping^7^.

During object manipulation, when lifting and holding a small object using the precision grip, i.e. between the thumb and the opposite fingers, cues about the skin-surface friction allow secure object manipulation without slip^5,22,23^. When the surface of the object change between trials, force adjustments occur very early during the object-skin interaction, i.e. within the contact and loading phases. More precisely, the balance between the grip and the lifting force is adjusted to the friction coefficient of the object’s surface^5^: the more slippery the surface, the higher the further grip force adjustment for the same load force. Slips, which can be defined as sudden small decreases in lifting force, also occur later in the interaction, i.e. during the holding phase. After such slips, an increase in grip force was observed within 75 ms, with a safety margin that prevents future slippages^5^.

Frictional cues activate the mechanoreceptors located in the glabrous skin of the hand. Mechanoreceptors within the median or ulnar nerves transduce these physical cues into meaningful nerve signals that inform the central nervous system about the surface’s identity or the force adjustments needed to secure a grip. The afferent responses to frictional cues have been described in only one study; in the context of object lifting tasks^1^. It was proposed that the frictional characteristics of the surface are encoded in FAI fibers, since the early phase of contact with the object, in an inverse fashion, where more slippery surfaces evoked greater responses. Slips occurring during the holding phase elicited responses in FAI, FAII and SAI units. Responses varied from single impulses to bursts (up to 10 impulses) and were elicited with a delay of ca. 10 ms after the onset of the slip. SAII units showed no specific responses to those slip events. In this study, it was concluded that FAI fibers might transduce specific information about the frictional properties of the surface touched. This is however intriguing when the basic view regarding human mechanoreceptor properties suggest that forces are encoded in the slow-adapting (SA) type of fibers, with SAI fibers conveying information about pressure, and SAII about lateral skin stretch, whereas the fast-adapting (FA) type are assumed coding for vibratory aspects related to touch^24^. Possibly, the textured surfaces (silk, suede and sandpaper) elicited vibrations that excited FA fibers^25^.

In the present study we used an innovative tactile stimulator, the Stimtac to reduce same-surface friction without adding lubricants^26^. More precisely, the technology is thought to create a thin layer of air (squeeze film effect) and an intermittent contact between the finger and the surface, reducing its friction level^27–29^. Both the amount of the friction reduction and the time taken to achieve the reduction can be manipulated to alter the perceived sharpness of the transient^17,30,31^. Phases of increase/decrease in friction can also be alternated to create time varying friction patterns that emulate real world textures such as gratings. Thus, we will be able to characterize the afferent responses that are evoked by pure lateral force modulation. The frictional transients elicited here are in amplitude comparable to small slips, while the changes in friction levels are comparable to a change in the friction coefficient of a surface.

## Methods

### Participants and general procedure

5 women aged 21–28 years, all right-handed, took part in the study. The study was approved by the ethics board of the University of Gothenburg (number S506-01) and was conducted according to the Declaration of Helsinki. Participants were paid for their participation. They all provided written informed consent. After participants had been comfortably seated in a reclining chair, with their left arm immobilized using a vacuum cast, microneurography recordings of single afferent units were obtained from low-threshold mechanoreceptors located in the volar part of their left hand. For each single afferent unit isolated and identified, a range of stimulations was delivered over the receptive field of the unit using the Stimtac device. In total, 8 tactile afferents were studied including 2 FAI, 1 FAII, 2 SAI and 3 SAII units.

### Microneurography recordings

Nerve impulses were recorded from single afferent fibers in the median nerve, which innervates the volar part of the thumb, index, middle fingers and the radial side of the ring finger. Details regarding the localization and recording procedure have been described in previous studies^32^. Briefly, the nerve was localised using an electrical search procedure and a recording electrode (0.2 mm shaft diameter; FHC, Inc., Bowdoin, ME, USA) was inserted into the median nerve approximately 80 mm proximal to the elbow. The nerve signal was amplified, band pass filtered (0.2–4 kHz) and displayed on-line through loudspeakers and on computer screens. The signal was digitized at 16 kHz using a Power 1401 and the Spike2 software (CED Ltd., Cambridge, UK). The recording electrode was then adjusted until the activity of a single afferent could be recorded. At this stage, the experimenter continuously stroked the volar aspect of the participant’s fingers, using their own fingers, in order to evoke nerve responses and isolate unitary recordings from individual mechanoreceptive afferents.

Afferents were classified as fast or slow adapting type I or II based on their response to sustained indentation, and on the size of their receptive field, using calibrated monofilaments. SA units responded continuously to sustained stimulation. SAII units were further distinguished from SAIs by their spontaneous activity, large receptive fields and responsiveness to remote lateral skin stretch. Both types of FA units responded to the onset and offset of continuous stimulation. In addition, FAIIs had larger receptive fields, and responded to remote stimulation and blowing over the skin.

### Devices

**Figure 1:**
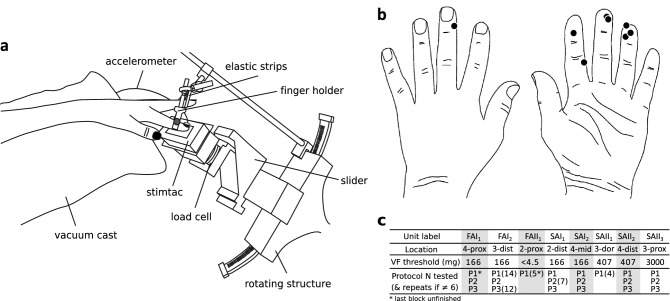
Experimental set-up and unit locations on the hand. (**a**) Stimtac unit mounted on the robot platform and its adjustable mount. (**b**) Locations of the centers of Receptive Fields (RFs) of the 8 mechanoafferent units evaluated in the paper. (**c**) Summary table of the unit characteristics, from top to bottom: unit label, location (2–3–4 for index-major-ring fingers; *prox* proximal, *dist* distal, *mid* middle, *dor* dorsal), Von Frey threshold, protocols performed, and number of repeats of blocks per unit.

A custom-built device, the Stimtac, was constructed by L2EP (University of Lille, France) to be mounted on a robot platform^33^. The Stimtac is a tactile stimulator that uses piezoelectric motors to deliver ultrasonic vibrations to reduce the friction of a surface^26,34,35^. The vibratory aspect itself is unperceived, as the frequency range (38 kHz) overrides the detection capacities of human mechanoreceptors. An increase in vibratory amplitude decreases the friction between the surface and the finger. The interface was comprised of an 1.2 mm aluminum plate covered with a thin polyvinyl chloride film with paper-like surface finishing (rugosity parameters: S_a_ = 1.2 µm, S_z_ = 6.9 µm), and an external controller for ultrasonic vibration of the plate (frequency range: 38 kHz, max amplitude: 1.3 μm). The amplitude of ultrasonic vibration was acquired at 3200 Hz by the acquisition toolchain. The plate was mounted on a robot platform^33^ to enable delivery of passive stimulations with controlled speed and normal force (see Fig. 1a). Forces were measured with a 6-axis load cell integrated to the robot platform (ATI Industrial Automation, Apex., NC, USA, bandwidth limited to 200 Hz) and oversampled at 800 Hz. The robot platform was also adjustable in height and angle. A finger holder was used to stabilize the finger position. It was attached at the level of the interphalangeal joint of the first phalanx. Elastic strips were used when necessary to support the other fingers. A tri-axial accelerometer (Prof. Veikko Jousmäki, Alto University, Brain Research Unit, Espoo, Finland; 500 Hz bandwidth) was also attached to the fingernail with double-sided tape, and was oversampled at 3200 Hz. All recordings and stimulus deliveries were controlled using Spike2. These signals were displayed on-line during the experiment.

### Skin stimulation using the Stimtac

At the beginning of each experiment, the Stimtac surface was cleaned with ethanol. Talcum powder was also applied on the finger to prevent mechanical vibrations sometimes observed during pre-tests and which were clearly identifiable using the nail mounted accelerometer. Once a unit had been isolated and characterized, the general procedure was to: (1) orient the robot platform parallel to the afferent receptive field whilst approximately 1 cm under the skin surface, (2) fix the finger with the finger holder, (3) apply talcum to the finger, (4) stick the accelerometer to the nail. (5) The robot then contacted the finger by moving up until achieving a normal force of 0.4 N, (6) moved in a lateral sliding motion in the ulnar direction at a constant speed of 20 mm/s, (7) and finally disengaged from the finger by moving down to home position. The entire robot motion (up-slide-down) was then repeated in the radial direction.

Three independent protocols referred below as P1, P2, and P3, were used to examine single-unit mechanoreceptor responses to transient changes in friction levels. Each block, here defined as repetitions of sliding motions of the robot over the finger, started with a pair of sliding motions without activation of the ultrasonic vibration (0 μm amplitude) in order to check for the base firing level of the afferent units. P1 was a step change in friction level that was modulated either from the base friction level caused by the surface to a lower friction level (amplitudes of ultrasonic vibration evaluated: 0.1, 0.75 and 1.2 μm), or reversely; allowing to evaluate two friction conditions, respectively a decrease or an increase in friction level. The rise time, which is the time taken by the device to reach a given amplitude of ultrasonic vibration, was maintained at a constant value of 5 ms. Each sliding motion lasted 2 s, and a block contained 14 sliding motions in total, which accounts for the mere combination of the distinct variables manipulated, i.e. [2 sliding directions × 2 friction conditions × 3 amplitudes of vibration + 2 sliding motions without ultrasonic vibration]. In P2, we manipulated the rise time (0.5, 2.5, 5, 10, 20 or 40 ms to reach 1.2 μm), which affects the sharpness of the frictional transient. Three successive stimulations were delivered per sliding motion with at least 50 ms delay between stimulation offsets. Here, a sliding motion lasted 1 s. Each block contained 10 sliding motions for a total of 24 stimulations ([2 sliding directions × 2 friction conditions × 6 rise times + 2 sliding motions without ultrasonic vibration]). In P3, friction was modulated by alternating phases of increase/decrease in ultrasonic vibration in order to simulate grating-like patterns. Seven simulated gratings were used, with spatial periods of 3200, 1600, 800, 400, 240, 160 and 80 µm (corresponding to fundamental frequencies of 6, 12, 25, 50, 85, 125, and 250 Hz). The amplitude of ultrasonic vibration was periodically turned on and off up to 1.2 μm, the rise time was set to 5 ms, and a sliding motion lasted 1 s. Each block contained 16 sliding motions in total ([2 sliding directions × 7 spatial periods + 2 sliding motions without ultrasonic vibration]. The three protocols were always delivered in the order P1, P2, and P3. We aimed to repeat blocks at least 6 times per units when recording stability was sufficient (see Fig. 1c for details). Stimulations were presented in semi-randomised order for all protocols. As an example for the randomisation procedure; in P1, the amplitude of ultrasonic vibration was kept similar per pair of sliding motion (ulnar and radial), amplitude values changed with each new pair of sliding motion, and the friction condition (increase or decrease) changed with each repetition of the same amplitude condition. Before starting the microneurography procedure, participants were familiarized to the protocols by trying each one once on the index fingertip.

### Variables measured

Recorded nerve impulses were inspected off-line on an expanded time scale using Spike2. Nerve impulse trains were accepted for subsequent analyses only if they could be properly validated as originating from a single afferent^32^. All recordings (amplitude of ultrasonic vibration, forces, acceleration, and nerve signal) were exported from Spike2 and analyzed in Matlab. Because the normal force was maintained constant by the robot platform, the lateral force was used as the main measure of the friction level in all protocols.

For all protocols (P1, P2, P3), the physical variables, i.e. the amplitude of ultrasonic vibration (μm), the profiles of lateral force (N) or the acceleration profiles (m s^−2^), were plotted along with the neural data, i.e. raster plots (the time at which spikes are elicited as a function of each repeated stimulation), at individual or group level. For all latency measurements, the onset value (zero) was defined as the moment preceding the first change in the amplitude of ultrasonic vibration. In addition, in P1, we measured the *friction coefficient* of the surface during trials with no Stimtac modulation, during the stable phase of the stimulation, as the ratio of the averaged lateral and normal forces. To characterize the changes occurring in friction level, we measured, *the change in lateral force *(*N*) as the absolute difference in lateral force between the stimulation phase and the baseline on 600 ms epochs (600 ms before onset, and 100–700 ms after onset). To characterize unit firing, *Peri-Stimulus Time Histograms* (*PSTH*, i.e. histograms of onset-to-spike latencies) were computed in 10 ms bins and plotted on the top of each raster plot. From the PSTH, we derived the peaks’ bin positions after combining all latencies from the three conditions of ultrasonic vibration. Z-scores, i.e. (average count of latencies – count of latencies at peak)/standard deviation, were calculated to ensure peak significance (> 3 s.d.). The *proportion of spikes at peak* was computed by dividing the count of latencies at peak by the total count of latencies obtained for the three conditions. We also measured the *instantaneous firing rate* (*Hz*) as the average of the inverse of the inter-spike intervals; the *difference in number of spikes* between the stimulation phase and the baseline (600 ms epochs as previously); and the *first spike latency* (*ms*) within a 100 ms time window. In P2, to characterize unit firing, we measured the *first spike latency* (*ms*) within a 50 ms time window; its *standard deviation* (*ms*); and the *proportion of stimulations with spikes *(%, percentage of stimulations for which at least a spike was elicited). In P3, to verify whether the skin was stimulated at the expected frequencies in spite of the limited bandwidth of the load cell to 200 Hz, power spectrum of the force and acceleration measurements were computed. Histograms of firing frequencies were also computed in Hz as histograms of the inverse of the latencies obtained between successive spikes.

### Statistical analyses

Regarding unit firing characterization, analyses were always done separately in the ulnar and radial direction due to the known existence of directional preferences in firing (especially for the SA type). More generally, statistical analyses were conducted in SPSS following standard procedures. To compare a mean to a norm, the Student test (*t*) was used. When repeated measures were compared, if the conditions of normality and sphericity were respected (α > 0.10), ANOVA for repeated measures (F) were used along with LSD post hoc tests when required. If the condition of sphericity was violated, the Greenhouse–Geisser correction was applied. In the absence of normality, the non-parametrical Friedman test (χ^2^) was used as the main test, and the Wilcoxon signed-rank test (z) was used for post hoc testing with Bonferroni correction. Pearson’s correlations were also computed when needed. Default alpha was 0.05.

## Results

### Database

In total, 8 units were stimulated including 2 FAIs, 1 FAII, 2 SAIs and 3 SAIIs (see Fig. 1b for an illustration of the receptive field (RF) locations of the units on the hand). Mechanical thresholds measured with Von Frey (VF) hairs were lowest for the FAII unit (< 4.5 mg), intermediate for the FAIs and SAIs (166 mg), and, were higher for the SAIIs (407–3000 mg), see Fig. 1c for details. The protocol blocks were repeated 4 to 14 times depending on the unit tested (see Fig. 1c for details), for a total of 627 stimulations for P1, 888 stimulations for P2, and 588 stimulations for P3. The stimulation was applied to the center of the receptive field in all except two units for which the center of the receptive field could not be reached (SAII_1_ and SAII_2_) due to limitations in positioning the platform. These two units still responded vigorously to stimulation 2–3 mm away from the center. FAII_1_ was tested with P1 only, with parameters slightly different as compared to the other units; i.e. with normal force of 0.7 N, with minimal amplitude of vibration 0.4 μm, and, with sliding motion duration of 1 s, and, without using talcum powder (because no mechanical vibration of the set-up was observed during the pre-test. Note that talcum was then used systematically). The friction coefficient of the surface was on average equal to 0.38 (s.d. = 0.07; N = 7), when excluding the results of FAII_1_, for which the friction coefficient was larger (mean = 0.99) due to the difference in the force parameters initially set. The robot platform adequately achieved the expected normal force of 0.4 N (mean = 0.40; s.d. = 0.01 when excluding the results from FAII_1_).

### Protocol 1: step in friction level

**Figure 2:**
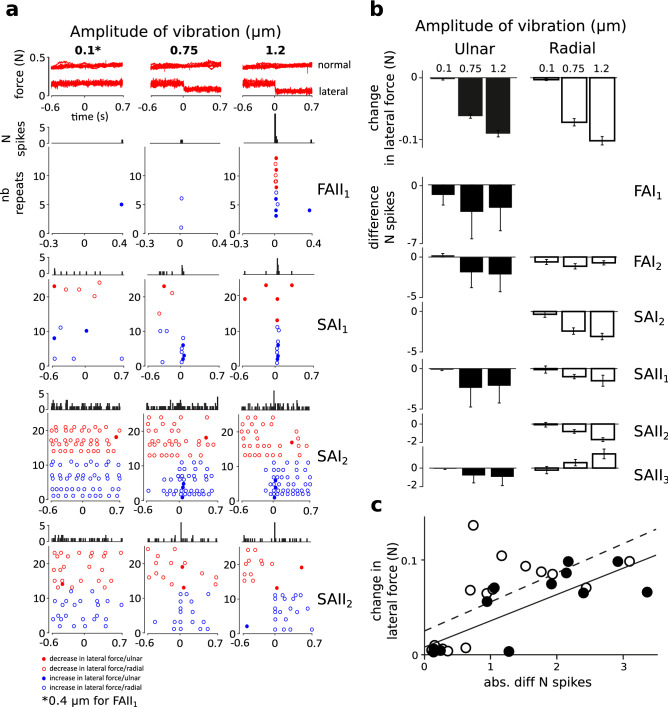
Step in friction level. (**a**) Top panel, superimposed recordings of normal and lateral force for the three amplitudes of ultrasonic vibration tested (0.1 μm, 0.75 μm and 1.2 μm). The action of the device from t = 0 effectively reduced the lateral force on the side of the modulation for the two larger amplitudes. Panels below show PSTHs (10 ms bins) and raster plots of unit firing for four representative units (FAII_1_, SAI_1_, SAI_2_ and SAII_2_). Colours illustrate the friction condition, i.e. decrease in friction level over time (red) or increase (blue). Dot filling represents the sliding direction, with filled dots for the ulnar direction and unfilled dots for the radial direction. Note that the sequences of repeats have been grouped per friction condition in the plots to improve their readability; hence they do not reflect the randomization sequences. For each unit, all repeats are shown. Repeats are left empty when no spikes were elicited, which highlights the scarcity of the responses to the stimulation. FAII_1_ and SAI_1_ showed time-locked responses to the moment of change in friction. SAI_2_ and SAII_2_ showed the same effect, revealed by the PSTH, as well as a reduction in the number of spikes with friction reduction (stable phase of the stimulation). (**b**) Top panel, average change lateral force (i.e. including the conditions of increase or decrease in friction) measured separately for the two sliding directions, and for the three amplitudes of ultrasonic vibration tested for the 8 units. Panels below show the average difference in number of spikes (between the baseline vs. stimulation phase) for each unit. The error bars represent the standard error of the mean. (**c**) Correlations between the change in lateral force vs. the change in the number of spikes are shown at group level, in the ulnar (black dots, black regression line); and radial directions (unfilled dots, dashed regression line).

As illustrated in Fig. 2a,b-top, the lateral force was effectively reduced by the effect of the Stimtac (F(3,21) = 265.56, p < 0.001), while the normal force was not affected. There was also a main effect of the sliding direction (F(1,7) = 7.92, p < 0.05), with the change in lateral force being slightly smaller in the ulnar direction (mean = − 0.038 N) compared to radial (mean = − 0.044 N). Pairwise differences were all significant (p < 0.05; pairwise LSD post hoc tests).

Raster plots observed at baseline (no friction reduction, amplitude of vibration = 0 μm) revealed sparse unit firing (mean instantaneous firing rate 0–8 Hz), with only 1 spike or less per sliding motion elicited in two units (FAII_1_ and SAI_1_), and with spikes elicited in only one direction for 3 units (FAI_1_, SAI_2_ and SAII_2_). Those units firing with 1 spike or less at baseline (FAII_1_ and SAI_1_), showed specific single responses (1 spike) to the frictional transient in the test conditions (see the raster plots in Fig. 2a). In the condition of maximal change in friction level (1.2 μm), the average first spike latency was equal to 14 ms (standard deviation 7 ms) for FAII_1_, and to 36 ms (s.d. = 7 ms) for SAI_1_. The single spike response was observed independently of the friction condition (increase or decrease in friction), and seemed to occur more often with higher amplitude of change in friction level (see Fig. 2a, FAII_1_ and SAI_1_). The PSTHs confirmed that the single spike response observed right after the frictional transient also occurred in other units (all except FAI_2_ and SAII_1_, z-score < 3 s.d, see for example SAI_2_ and SAII_2_ in Fig. 2a), with peaks obtained between 10 to 30 ms bins (mean = 20 ms; s.d. = 9 ms). This result was also confirmed by a group analysis, in which we compared the proportion of spikes at peak as a function of the amplitude conditions (F(1.13,7.93) = 13.992, p < 0.005), with however no differences obtained between the two larger conditions of ultrasonic vibration (0.75 and 1.2 μm, LSD post hoc, ns). This suggests that the proportion of spikes at peak might be used as a neural code for the amplitude of the frictional transient.

For those units firing during platform movement, firing was clearly reduced with friction reduction, as illustrated in the raster plots of SAI_2_ and SAII_2_ in Fig. 2a. The effect was more pronounced for the maximal amplitude of ultrasonic vibration tested (1.2 μm), where units often ceased firing entirely. Differences between the stimulation phase and the baseline were significant in at least one direction for most units (see Fig. 2b-bottom, FAI_1_-ulnar: F(2,22) = 0.57, ns; FAI_2_-radial: χ^2^ = 3.34, ns; ulnar: χ^2^ = 9.80, p < 0.01; SAI_2_-radial: (2,22) = 14.07, p < 0.001; SAII_1_-radial: F(1,14) = 7.56, ns, ulnar: χ^2^ = 9.80, p < 0.01; SAII_2_-radial: χ^2^ = 15.62, p < 0.001; SAII_3_-radial: F(2,22) = 4.51, p < 0.05, ulnar: χ^2^ = 3.23, ns). For SAII_3_, the inverse effect was obtained in the radial direction: the number of spikes increased the larger the friction reduction. To confirm the effect of change in friction level at group level, we also computed the correlations between the absolute friction reduction and the absolute difference in the number of spikes, which were significant both in the radial (R = 0.62, p < 0.05) and ulnar directions (R = 0.78, p < 0.005) (Fig. 2c), hence confirming the existence of a positive linear relationship between the physical and neural aspects related to a change in friction level.

### Protocol 2: manipulation of the rise time

**Figure 3:**
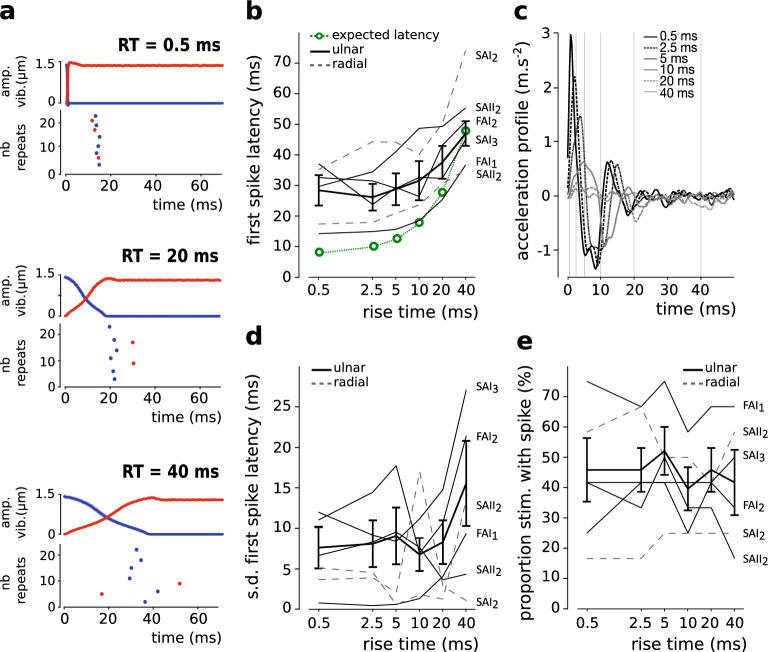
Rise time manipulation. (**a**) Profiles of ultrasonic vibration (average and s.d. traces), and the corresponding raster plots are shown for FAI_1_ for three rise time conditions (0.5, 20 and 40 ms). Colours illustrate the friction condition, i.e. increase in friction over time (which corresponds to a decrease in ultrasonic vibration value, blue) or decrease (red). Dot filling represents the sliding direction, with filled dots for the ulnar direction and unfilled dots for the radial direction. Note that the unit presented FAI_1_ does not fire in the radial direction. (**b**) First spike latency as a function of the rise time conditions. Individual traces (ulnar and radial) and group results (ulnar only, N = 4). The error bars represent the standard error of the mean (s.e.m). In green, an estimation of the expected first spike latency. (**c**) Group acceleration profile. Individual acceleration curves were normalized as a function of the friction condition (increase or decrease) and of the sliding direction. They were then averaged for each participant, and are here presented as an average obtained for the group (N = 6). The grey grid in background shows the expected stimulation time offsets. Acceleration peaks pointing upwards are revealed especially for the short rise time conditions, at approximately the expected moment, and, their amplitude seems to increase the shorter the rise time. Downwards peaks are also seen around 10, 20 and 40 ms. This profile confirms that the programmed events occurred at the expected moments in time. (**d**) Standard deviation of the first spike latency as a function of the rise time conditions. The error bars represent the s.e.m. (**e**) Proportion of stimulations with spike as a function of the rise time conditions. The error bars represent the s.e.m.

Figure 3a shows the average and s.d. traces of the recorded amplitude of ultrasonic vibration, as well as the corresponding raster plots, for three of the rise time conditions tested (0.5 ms, 20 ms, 40 ms) in one representative unit (FAI_1_). Here again, units responded sparsely to the stimulation. Most units responded in only one direction (i.e. ulnar or radial), with 0 or 1 spikes per stimulation (2 spikes were elicited for only one stimulation out of 888 stimulations). Note nevertheless, here again, that these single spikes were elicited time-locked to the stimulation (see Fig. 3a). This single spike profile was seen in two units (FAI_1_ and SAII_2_). The latency between the stimulation and the spikes seemed disproportionally longer for the shorter rises as compared to the longer ones (see Fig. 3a). The timing of the spikes also seemed to be more variable for the longer rise times. Accordingly, to characterize unit firing at group level, we measured the first spike latency, its standard deviation, and the proportion of stimulations with spikes. Group level analyses were conducted in the ulnar condition only (N = 4), as only two units fired enough in the other direction (i.e. with less than 2 spikes in total in some conditions). Individual unit responses and/or averages are nevertheless shown in the plots for the two directions (Fig. 3b, d, e). The first spike latency was significantly longer with longer rise time in the ulnar direction (F(5,15) = 10.113, p < 0.001, Fig. 3b), confirming the existence of a relationship between the rise time modulation and the first spikes elicited. We also compared the first spike latencies to expectations, which here includes the delays of neural conduction, i.e. 8 ms for an estimated receptor to recording location distance of 50 cm, and for an estimated conduction velocity of 40 m s^−1^ for Aβ fibers^36^ (see the green line in Fig. 3b). Differences to expectations were significant in the two shorter RT conditions only (RT_0.5_: t(3) = 3.741, p < 0.05; RT_2.5_: t(3) = 3.273, p < 0.05; RT_5_: t(3) = 2.774, ns; RT_10_: t(3) = 1.903, ns; RT_20_: t(3) = 1.526, ns; RT_40_: t(3) = − 0.60, ns), which confirms larger latencies than expected (16 ms extra on average) for the two shorter RTs. In order to re-check for the temporal precision of the stimulus delivery, we plotted the acceleration profile of the group (N = 6), after normalizing the individual acceleration traces as a function of the friction condition (increase or decrease) and the sliding direction, i.e. so that the first peak pointed upwards whatever the condition, see Fig. 3c. Positive and negative acceleration peaks were revealed at the expected moments during the stimulation, with positive peaks reflecting the onset of the stimulation especially for the short rise time conditions, and negative peaks reflecting the offset of the stimulation especially for the two longer rise time conditions. Also note that the amplitude of the positive peak was larger the shorter the rise time, confirming that stimulations were sharper the shorter the rise time. Altogether, the group acceleration profile confirms that the stimuli were provided as expected, i.e. without delays that could explain the ca. 15 ms of extra latency. The variability of the moment at which the spike is elicited—measured here as the standard deviation of the first spikes latencies-, and the possibility that single spikes occur more often for sharper stimulations—measured here via the proportion of stimulation with 1 spike, did not change with the rise time conditions (F_var_(5,15) = 2.329, ns, see Fig. 3d; F_prop_(5,15) = 1.333, ns, see Fig. 3e). Taken together, these results suggest that the sharpness of frictional transient, controlled here through its rise time, is encoded in the first spike latency, with however latencies longer than expected for the shorter rise times.

### Protocol 3: gratings

**Figure 4:**
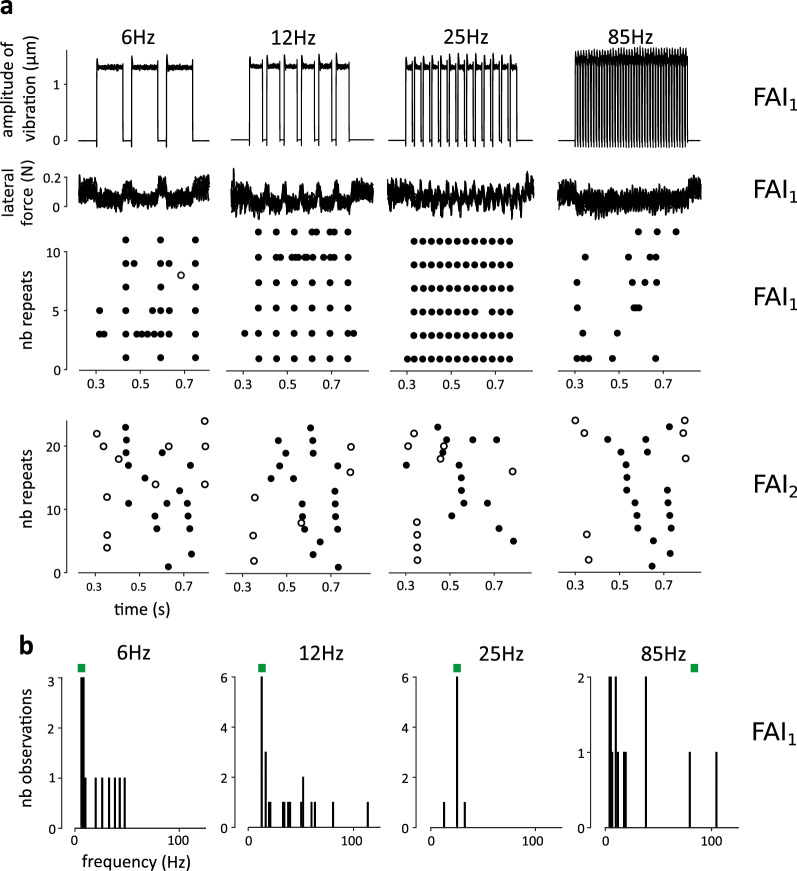
Gratings. (**a**) Profiles of ultrasonic vibration, profiles of lateral force, and the corresponding raster plots are shown for FAI_1_ at four distinct frequencies of stimulation: 6, 12, 25 and 85 Hz. Filled/unfilled dots indicate ulnar/radial direction of sliding. Firing followed the frequency of stimulation in a time-locked fashion between 6 to 50 Hz for this unit. Raster plots are also shown for FAI_2_ at the same frequencies, where there was no frequency following. (**b**) Histogram of firing frequencies obtained for each condition are shown for FAI_1_.

Figure 4a shows the profiles of ultrasonic vibration used to create the grating-like patterns, the corresponding changes occurring in lateral force, and raster plots for two representative units FAI_1_ and FAI_2_. Power spectra of the force and acceleration signals confirmed that the skin was effectively stimulated up to 250 Hz, with peaks in either the force or the acceleration signals observed at the expected frequencies (not shown).

In most units, time-locked patterns of firing were elicited in strict relationship to the delivered stimuli (Fig. 4a). This effect, visible to the naked eye was also confirmed by histograms of firing frequencies, which showed distinctive peaks at the programmed frequencies (Fig. 4b). Unit firing followed the periodicity of the stimulation up to 6 Hz for all units (N = 6), up to 12 Hz for SAII_2_, and up to 50 Hz for FAI_1_. Note however that for FAI_2_, the firing frequency was equal to 6 Hz in all conditions, but was independent of the programmed pattern of stimulation (Fig. 4a). Overall, the results suggest that the Stimtac can simulate spatially structured textures that modulate the firing patterns of low-threshold glabrous skin mechanoreceptors in a time-locked fashion.

## Discussion

In the present study, we used the Stimtac device to generate transient reductions in friction in order to study these effects on the cutaneous afferents of the hand without changing the surface properties. Our results mainly revealed that (1) afferent fibers encoded frictional transients with a single spike, and, irrespective of the direction of the change (increase or decrease in friction), and that, (2) the reduction in friction level reduced the number of spikes during the stable phase of the modulation. The occurrence of single spikes and the first spike latency coded for the amplitude of the frictional transient and for its sharpness respectively. Both effects were found for all afferent classes (SA or FA), which suggest that frictional cues are not encoded by a specific unit class. These results extend and challenge the findings of the unique previous study focused on the coding of friction-related events in human low-threshold mechanoreceptors^1^.

The first effect obtained in the present study, i.e. that single spikes are elicited in response to frictional transients partially replicates the findings of Johansson and Westling^1^. Indeed, they found that object slippage elicited firing in the form of single impulses or bursts (1–10 impulses), with peak frequencies up to ca. 300 imp s^−1^, and with a delay of ca. 10 ms after the onset of the slip in FAI, FAII and SAI units. In the present study, we did not observe burst responses to the delivered frictional transients, we only observed single spikes, which were elicited very systematically ca. 15 ms after the slip (when subtracting the delays due to neural conduction). Several arguments could explain this difference. First, the decrease in friction obtained during natural slips when holding an object could have been of larger amplitude, hence increasing the spike to slip ratio from 1 (as seen in the present study) to several spikes. Indeed, in the present study, the maximal achievable change in lateral force was ± 0.1 N given the normal force used, hence a force ratio of 0.25 (0.1/0.4 N, see Fig. 2b). In the study of Johansson and Westling^1^, slips are described as small and their amplitude is not systematically reported. From the example shown in their figure (eight), one can estimate force ratios as varying between 0.2 to 1 (measured as the amplitude of change in lateral force divided by the normal force). Those were then probably larger than those programmed in the present study. Second, one could consider that due to the ecological nature of object manipulation, a succession of frictional transients rather than a net change in friction occurred in their study. In the present study (P2), the firing response due to pure frictional transients demonstrated a minimum latency. Indeed, taking into account the delays due to conduction, the spikes were elicited ca. 15 ms after the stimulation. This minimum latency was not explained by a lack of temporal precision of the device, as shown with the acceleration profile obtained for the group (Fig. 3c). Accordingly, this result could reflect that the skin needed time, ca. 15 ms, to react to the frictional transient, i.e. to stretch, or to successively stretch and relax its viscoelastic tissues. Further studies using tribometers or high-speed cameras with higher temporal resolution than the load cell used in the present study are needed to explore further what is the exact relationship between the mechanical events occurring along with frictional transients and the related afferent firing. Nevertheless, if one considers the existence of such minimal latency, and considering a 1:1 spike to slip ratio, then one should not expect to obtain firing rates superior to ca. 70–100 imp s^−1^ in response to pure frictional transients. This prediction raises the possibility that the bursts obtained in the study of Johansson and Westling^1^ could have reflected the vibratory properties of their stimulus rather than their frictional properties. Indeed, in their study, textured surfaces (suede, silk or sandpaper) were attached to the object to lift and hold. Those textures, which vary regarding their friction coefficient^37^, may have also varied according to their roughness, and as such high-frequency skin vibrations may have been generated when interacting with the skin^25^. In our study, we used the Stimtac and a very smooth surface to generate frictional transients without the associated textural vibrations^38^. Note that this assumption is also supported by an effect they reported, i.e. bursts frequencies occurring along with the slips in the holding phase increased with the roughness of the materials rather than with their slipperiness (ca. 200 imp s^−1^ with suede or silk, and, ca. 400 imp s^−1^ with sandpaper).

Another result obtained in the present study, but not found in the study of Johansson and Westling^1^, is that SAII units consistently responded to friction modulation. SAII_2_ and SAII_3_ responded to the frictional transients (peaks in PSTH), in P1. SAII_2_ showed a very distinctive pattern (similar to what presented for FAI_1_ in Fig. 3a) in P2. Here again, the difference obtained could be explained by the differences in the surfaces used: rough-vibratory textured surface vs. a smooth-sticky one. Indeed, a stickier surface could have provided enough skin stretch to elicit significant firing in SAII units, which, as confirmed in the present study demonstrate higher mechanical thresholds to fire compared to the other types of low-threshold mechanoreceptors^39^. This result supports the findings of other studies showing that SAIIs, and especially SAII-nail units (as SAII_2_ in the present study) can demonstrate high sensitivity to changes in lateral force^40^, and so forth contribute to the neural coding of frictional slips. More generally, this result reflects that all unit types in the present study responded to frictional transients, which suggests that frictional slips are encoded in an unspecific manner. Taken together, the results obtained in the present study regarding the frictional transients support the view that single spikes can provide a neural code (occurrence and first spike latency) rich, fast and salient enough for the brain, especially if represented in a large population of receptors, to trigger automatic motor responses within a ~ 75 ms loop to support precision grip^30,41^.

The finding that frictional slips are encoded in all unit types challenges the current view that the FA1 type might be the main unit type coding for the level of friction of a surface. Indeed, in the study of Johansson and Westling^1^, FAI units responded at initial contact, i.e. when the fingers pressed on the object without moving laterally. The authors proposed that units might have encoded the surface friction since this very early phase, hence providing relevant information for later adjustments. In addition, in this very early moment of the object/finger interaction, units fired more with more slippery textured surfaces (i.e. more firing for silk as compared to sandpaper), hence providing a neural code very specific to the slippery status of the stimulation. The view that the FA1 type encode the slipperiness of a finger-surface interaction is also supported by a microneurography study of Macefield, et al.^42^, in which dynamic ramp and hold stimulations with precise control of the load force and rate of change were applied to the receptive fields of units. In this study, the surface was covered with suede. FAI units especially fired during the ramp phases, fired for a longer period of time for slower ramps, but were silent during the hold phases. FAII units responded similarly to what described in the present study, i.e. to the transition between the ramp or hold phases, but only when the rate of change was high. Other unit types responded both to changes in the grip or load forces in a way less specific compared to FAs. Taken together, the authors suggested that FA1 units provided the more relevant responses to code the level of friction. None of these results were replicated in the present study. The FA1 type did not show any specificity. Here again, the main difference between those studies and the present study lies on the surfaces used (rough vs. sticky). Here, one should also consider the possibility that FA1 might have fired in response to a specific physical variable reflecting an interaction between the frictional and vibratory properties of a textured surface (perhaps its slipperiness), rather than one of the two independent variable, i.e. textural vibration or friction level, as proposed in a recent behavioral study^43^.

A second effect, novelty of the present study was that decreasing (or increasing) the friction level reduced (or increased) the number of spikes during the stable phase of the modulation. This finding is in line with the results of previous studies that used occlusive agents such as soaps or lubricants to reduce the friction of surfaces stimulating the skin, and observed a decrease in the intensity of the evoked perception^12,14^. Hence, this suggests that the Stimtac similarly acted as an occlusive agent. Although the FAII tested in P1 did not fire during the stable phase of the stimulation, both the SA and FA types responded similarly to the friction reduction, suggesting that friction reduction is also encoded irrespective of the unit type. Interestingly, SAII_3_ showed the inverse tendency in one direction, i.e. an increase in the number of spikes with friction reduction. This result confirms the known result that SAIIs have directional preferences^36,39,40^. It could actually also reflect a specificity of this unit type to provide a neural code of the slipperiness dimension.

One should finally consider the potential of the Stimtac to control unit firing with a high degree of temporal precision. Indeed, as revealed in P3, in most units, firing was elicited in strict relationship to the delivered stimuli. This type of stimulation may be useful to researchers interested in how temporal regularities are represented at central levels^44,45^. One can also note that time-locked responses were obtained only up to 50 Hz for an FAI in the present study. As discussed earlier, this effect could be specific to frictional modulation, i.e. the skin may need time to react to each frictional transient, i.e. to stretch, or to successively stretch and relax its viscoelastic tissues, hence there may be a limit boundary to which this time-locked effect may be elicited (around 60 Hz if one assumes that the skin needs at least 15 ms to stretch and relax its tissues). This result may also simply reflect that the distinct unit types are sensitive to distinct frequency ranges of stimulation, with the SA type assumedly following temporal modulation in the low frequency ranges (< 10 Hz), compared to the FA type (around 30 Hz for the FAI type, and > 100 Hz for the FAII type)^46,47^. Further testing is thus needed to evaluate the previous interpretations.

To conclude, and, considering the consistency of our results over the three protocols tested in spite of the paucity of data presented, our results suggest that friction levels and frictional transients are encoded in all unit types. Reducing the friction level reduces the number of spikes elicited. Frictional transients are transduced into single spikes, through the *spike occurrence* reflecting their amplitude and the *first spike latency* reflecting their sharpness. We propose that the latter neural code might be salient enough to trigger the automatic responses needed for precision grip adjustments. The unspecific nature of friction coding also promotes this variable as a relevant parameter to mimic for use in prosthetics, i.e. to potentially recreate the slips engaged in natural conditions of object manipulation through the use of electrical stimulation to activate afferent fibers.

## References

[1] Johansson RS, Westling G (1987). Signals in tactile afferents from the fingers eliciting adaptive motor responses during precision grip. Exp. Brain Res..

[2] Lederman SJ, Klatzky RL (1993). Extracting object properties through haptic exploration. Acta Psychol. (Amst.).

[3] Jansen SE, BergmannTiest WM, Kappers AM (2013). Identifying haptic exploratory procedures by analyzing hand dynamics and contact force. IEEE Trans. Haptics.

[4] Callier T, Saal HP, Davis-Berg EC, Bensmaia SJ (2015). Kinematics of unconstrained tactile texture exploration. J. Neurophysiol..

[5] Johansson RS, Westling G (1984). Roles of glabrous skin receptors and sensorimotor memory in automatic control of precision grip when lifting rougher or more slippery objects. Exp. Brain Res..

[6] André T, Lévesque V, Hayward V, Lefèvre P, Thonnard J-L (2011). Effect of skin hydration on the dynamics of fingertip gripping contact. J. R. Soc. Interface.

[7] Gueorguiev D, Bochereau S, Mouraux A, Hayward V, Thonnard J-L (2016). Touch uses frictional cues to discriminate flat materials. Sci. Rep..

[8] Delhaye B, Barrea A, Edin BB, Lefèvre P, Thonnard J-L (2016). Surface strain measurements of fingertip skin under shearing. J. R. Soc. Interface.

[9] Delhaye B, Lefevre P, Thonnard J-L (2014). Dynamics of fingertip contact during the onset of tangential slip. J. R. Soc. Interface.

[10] Smith AM, Scott SH (1996). Subjective scaling of smooth surface friction. J. Neurophysiol..

[11] Yoshioka T, Bensmaïa SJ, Craig JC, Hsiao SS (2007). Texture perception through direct and indirect touch: An analysis of perceptual space for tactile textures in two modes of exploration. Somatosens. Mot. Res..

[12] Smith AM, Chapman CE, Deslandes M, Langlais J-S, Thibodeau M-P (2002). Role of friction and tangential force variation in the subjective scaling of tactile roughness. Exp. Brain Res..

[13] Klocker A, Wiertlewski M, Theate V, Hayward V, Thonnard J-L (2013). Physical factors influencing pleasant touch during tactile exploration. PLoS One.

[14] Nacht S, Close J, Yeung D, Gans EH (1981). Skin friction coefficient: Changes induced by skin hydration and emollient application and correlation with perceived skin feel. J. Soc. Cosmet. Chem..

[15] André T, Lefèvre P, Thonnard J-L (2009). A continuous measure of fingertip friction during precision grip. J. Neurosci. Methods.

[16] Pasumarty SM, Johnson SA, Watson SA, Adams MJ (2011). Friction of the human finger pad: Influence of moisture, occlusion and velocity. Tribol. Lett..

[17] Gueorguiev D, Vezzoli E, Mouraux A, Lemaire-Semail B, Thonnard J-L (2017). The tactile perception of transient changes in friction. J. R. Soc. Interface.

[18] Gerhardt LC, Strässle V, Lenz A, Spencer ND, Derler S (2008). Influence of epidermal hydration on the friction of human skin against textiles. J. R. Soc. Interface.

[19] Dinç O, Ettles C, Calabrese S, Scarton H (1991). Some parameters affecting tactile friction. J. Tribol..

[20] Smith AM (2010). Roughness of simulated surfaces examined with a haptic tool: Effects of spatial period, friction, and resistance amplitude. Exp. Brain Res..

[21] Lévesque, V. & Hayward, V. Experimental evidence of lateral skin strain during tactile exploration. in *Proceedings of EUROHAPTICS*, 6–9 (2003).

[22] Cadoret G, Smith AM (1996). Friction, not texture, dictates grip forces used during object manipulation. J. Neurophysiol..

[23] Cole KJ, Johansson RS (1993). Friction at the digit-object interface scales the sensorimotor transformation for grip responses to pulling loads. Exp. Brain Res..

[24] Vallbo ÅB, Johansson RS (1984). Properties of cutaneous mechanoreceptors in the human hand related to touch sensation. Hum. Neurobiol..

[25] Weber AI (2013). Spatial and temporal codes mediate the tactile perception of natural textures. Proc. Natl. Acad. Sci. U.S.A..

[26] Sergeant P, Giraud F, Lemaire-Semail B (2010). Geometrical optimization of an ultrasonic tactile plate. Sens. Actuators A.

[27] Wiertlewski M, Friesen RF, Colgate JE (2016). Partial squeeze film levitation modulates fingertip friction. PNAS.

[28] Vezzoli E (2017). Friction reduction through ultrasonic vibration. Part 1: Modelling intermittent contact. IEEE Trans. Haptics.

[29] Sednaoui T (2017). Friction reduction through ultrasonic vibration. Part 2: Experimental evaluation of intermittent contact and squeeze film levitation. IEEE Trans. Haptics.

[30] Gueorguiev D, Vezzoli E, Sednaoui T, Grisoni L, Lemaire-Semail B (2019). The perception of ultrasonic square reductions of friction with variable sharpness and duration. IEEE Trans. Haptics.

[31] Gueorguiev D, Vezzoli E, Sednaoui T, Grisoni L, Lemaire-Semail B (2017). Feeling multiple edges: The tactile perception of short ultrasonic square reductions of the finger-surface friction. IEEE World Haptics Conf. (WHC).

[32] Vallbo ÅB, Hagbarth KE (1968). Activity from skin mechanoreceptors recorded percutaneously in awake human subjects. Exp. Neurol..

[33] Oddo CM (2011). A mechatronic platform for human touch studies. Mechatronics.

[34] Amberg, M. *et al.* in *UIST'11, the 24th ACM Symposium on User Interface Software and Technology* 7–8 (ACM, 2011).

[35] Biet M, Giraud F, Lemaire-Semail B (2008). Implementation of tactile feedback by modifying the perceived friction. Eur. Phys. J. Appl. Phys..

[36] Nagi SS (2019). An ultrafast system for signaling mechanical pain in human skin. Sci. Adv..

[37] Dione M, Wessberg J (2019). Human 8–10 Hz pulsatile motor output during active exploration of textured surfaces reflects the textures' frictional properties. J. Neurophysiol..

[38] Adams MJ (2013). Finger pad friction and its role in grip and touch. J. R. Soc. Interface.

[39] Johansson RS, Vallbo ÅB, Westling G (1980). Thresholds of mechanosensitive afferents in the human hand as measured with von Frey hairs. Brain Res..

[40] Birznieks I, Macefield VG, Westling G, Johansson RS (2009). Slowly adapting mechanoreceptors in the borders of the human fingernail encode fingertip forces. J. Neurosci..

[41] Johansson RS, Birznieks I (2004). First spikes in ensembles of human tactile afferents code complex spatial fingertip events. Nat. Neurosci..

[42] Macefield VG, Häger-Ross C, Johansson RS (1996). Control of grip force during restraint of an object held between finger and thumb: Responses of cutaneous afferents from the digits. Exp. Brain Res..

[43] Fagiani R, Massi F, Chatelet E, Berthier Y, Akay A (2011). Tactile perception by friction induced vibrations. Tribol. Int..

[44] Moungou A, Thonnard J-L, Mouraux A (2016). EEG frequency tagging to explore the cortical activity related to the tactile exploration of natural textures. Sci. Rep..

[45] Kelly EF, Trulsson M, Folger SE (1997). Periodic microstimulation of single mechanoreceptive afferents produces frequency-following responses in human EEG. J. Neurophysiol..

[46] Freeman AW, Johnson KO (1982). Cutaneous mechanoreceptors in macaque monkey: Temporal discharge patterns evoked by vibration, and a receptor model. J. Physiol..

[47] Talbot WH, Darian-Smith I, Kornhuber HH, Mountcastle VB (1968). The sense of flutter-vibration: Comparison of the human capacity with response patterns of mechanoreceptive afferents from the monkey hand. J. Neurophysiol..

